# Phillyrin ameliorates influenza a virus-induced pulmonary inflammation by antagonizing CXCR2 and inhibiting NLRP3 inflammasome activation

**DOI:** 10.1186/s12985-023-02219-4

**Published:** 2023-11-13

**Authors:** Shanyu Zhang, Fengzhi Sun, Jinlu Zhu, Jianhong Qi, Wenjing Wang, Ziming Liu, Wenqian Li, Chuanguo Liu, Xuehuan Liu, Nonghan Wang, Xinyu Song, Dan Zhang, Dongmei Qi, Xiaolong Wang

**Affiliations:** 1 Shandong University of Traditional Chinese Medicine, Jinan, 250355 China; 2 Experimental Center, Shandong University of Traditional Chinese Medicine, Jinan, 250355 China; 3 Key Laboratory of Traditional Chinese Medicine Classical Theory, Ministry of Education, Shandong University of Traditional Chinese Medicine, Jinan, 250355 China; 4 Shandong Provincial Key Laboratory of Traditional Chinese Medicine for Basic Research, Shandong University of Traditional Chinese Medicine, Jinan, 250355 China; 5 Shandong Provincial Maternal and Child Health Care Hospital Affiliated to Qingdao University, Jinan, 250014 China; 6 Department of Pharmaceutics, China Pharmaceutical University, Nanjing, 211198 China

**Keywords:** Phillyrin, Influenza, Pulmonary inflammation, CXCR2, NLRP3

## Abstract

Influenza is an acute viral respiratory illness with high morbidity rates worldwide. Excessive pulmonary inflammation is the main characteristic of lethal influenza A virus (IAV) infections. Therapeutic options for managing influenza are limited to vaccines and some antiviral medications. Phillyrin is one of the major bioactive components of the Chinese herbal medicine *Forsythia suspensa*, which has the functions of sterilization, heat clearing and detoxification. In this work, the effect and mechanism of phillyrin on H1N1 influenza (PR8)-induced pneumonia were investigated. We reported that phillyrin (15 mg/kg) treatment after viral challenge significantly improved the weight loss, ameliorated pulmonary inflammation and inhibited the accumulation of multiple cytokines and chemokines in bronchoalveolar lavage fluid on 7 days post infection (dpi). In vitro, phillyrin suppressed influenza viral replication (Matrixprotein and nucleoprotein messenger RNA level) and reduced influenza virus-induced cytopathic effect (CPE). Furthermore,chemokine receptor CXCR2 was confirmed to be markedly inhibited by phillyrin. Surface plasmon resonance results reveal that phillyrin exhibits binding affinity to CXCR2, having a binding affinity constant (KD) value of 1.858e-5 M, suggesting that CXCR2 is a potential therapeutic target for phillyrin. Moreover, phillyrin inhibited the mRNA and protein expression levels of Caspase1, ASC and NLRP3 in the lungs of mice with H1N1-induced pneumonia.This study reveals that phillyrin ameliorates IAV-induced pulmonary inflammation by antagonizing CXCR2 and inhibiting NLRP3 inflammasome activation partly.

**Supplementary Information:**

The online version contains supplementary material available at 10.1186/s12985-023-02219-4.

## Background

Influenza is a fatal respiratory infectious disease. The excessive immune response caused by influenza infection may cause irreversible damage to the body [1, 2]. Influenza virus infection can lead to apoptosis or necrosis of tissue cells, which activates the immune system. The infected respiratory epithelial cells and vascular endothelial cells secrete cytokines and chemokines to recruit and activate various immune cells. Immune cells perform their respective functions to phagocytose pathogens and infected cells or secrete more cytokines, resulting in an expanded immune response [3]. An excessive immune response can bring serious consequences, such as immune cell infiltration and cytokine storm, which can result in pulmonary edema and may even cause death [4]. Excessive pulmonary inflammation is the main characteristic of lethal IAV infections.

Therapeutic actions for influenza are limited to vaccines and a few anti-viral drugs. However, the frequent mutation of influenza virus has limited the current treatment methods, and some studies have found that the cause of severe influenza is often an excessive immune response [5–7]. Therefore, antiviral therapy has been unable to meet our requirements, and a new way to resist influenza is urgently needed. Suppressing excessive immune responses to alleviate tissue damage caused by influenza virus infection could become an effective anti-influenza therapeutic strategy.

Phillyrin is one of the major bioactive components of the Chinese herbal medicine *Forsythia suspensa*, which has the functions of sterilization, heat clearing and detoxification [8–10]. We previously studied the efficacy of phillyrin in the treatment of influenza, and the results have shown that phillyrin could reduce the pathological inflammation of lungs in mice infected with FM1 strain of IAV and modulate the levels of various cytokines after influenza infection [11]. However, the ability of phillyrin to alleviate pulmonary inflammation caused by IAV infection and the mechanisms involved still require careful assessments.

This study sought to determine the effects and mechanisms of phillyrin on mice with pneumonia induced by H1N1 influenza. A H1N1-induced pneumonia mouse model was constructed, and the mice were treated with phillyrin following viral challenge. On 7 days post infection (dpi), the body weight, lung index, lung pathological changes, levels of multiple cytokines and chemokines in Bronchoalveolar lavage fluid (BALF) and expression levels of two viral proteins in the lungs were measured. The results indicated that phillyrin treatment obviously alleviated influenza-induced pulmonary inflammation. In vitro, phillyrin had potent antiviral effects against influenza infection in cell culture, which through suppression of matrixprotein (M) and nucleoprotein (NP) mRNA level as well as reduction of influenza virus-induced CPE. Furthermore, we elucidated the mechanism of phillyrin in alleviating influenza-induced pneumonia through RNA sequencing (RNA-Seq) analysis and molecular docking technology. We found that the cytokine-cytokine receptor interaction played an important role and C-X-C Motif Chemokine Receptor 2 (CXCR2) was confirmed as a promising therapeutic target. Moreover, phillyrin also inhibited the mRNA and protein expression levels of Caspase1, ASC and NLRP3 in the lungs of mice with H1N1-induced pneumonia. This study suggests that phillyrin may alleviate pulmonary inflammation in mice with viral pneumonia by reducing CXCR2 expression and suppressing NLRP3 inflammasome overactivation. It is hoped that our study will provide evidence to support the application of phillyrin in treating virus-induced pneumonia.

## Materials and methods

### Virus

Influenza A virus (PR8, H1N1) was provided by Shandong Provincial Collaborative Innovation Center for Antiviral TCM, China. The virus was placed at 37 °C and amplified in an 11-day-old chicken embryo for 2 days. Allantoic fluid was collected, centrifuged (1000 g, 10 min), and kept at − 80 °C. The 50% lethal dose (LD_50_) of the virus to BALB/c mice was detected by the Reed-Muench method.

### Cell experiments

Madin-Darby Canine Kidney (MDCK, maintained in our laboratory) cells were cultured in MDCK serum-free medium (Yocon, China) at 37℃ in an atmosphere containing 5% CO2. Cells were cultured at a density of 5 × 10^5^ cells per well in 6-well plates and allowed to grow for 24 h to a confluence of over 80%. MDCK cells were inoculated with IAV (PR8) at multiplicity of infection (MOI) 0.005. After 2 h adsorption, virus inoculum was removed and replaced with freshly prepared infectious media with phillyrin at different concentrations (100, 50, 25µM) or ribavirin at 10µM (as a positive control). Cell lysates were collected at 12 h post infection (hpi) and 24 hpi for subsequent experiments. Virus-induced CPE was recorded under microscopy at 36 hpi.

### Mouse experiments

Specific-pathogen-free (SPF) male BALB/c mice (6-week-old, weighing 16–18 g) were supplied by Beijing Vital River Laboratory Animal Technology (Beijing, China). All mice were housed in an environment at 20 ± 2% (humidity) and 22 ± 2 °C, and provided with a standard rodent diet and water. All experiments were conducted in compliance with the Regulations for the Administration of Affairs Concerning Experimental Animals in China, and were approved by the Animal Ethics Committee of the Animal Experiment Center of Shandong University of TCM following the regulations of the Jinan Administration Office of Laboratory Animals in Jinan, China. Before infection, the mice were subjected to respiratory anesthesia with 5% isoflurane. The animals were intranasally infected with a PR8 suspension (20 µL) at LD_50_ concentration diluted in phosphate buffered saline (PBS). Oseltamivir phosphate (a positive control drug) was given via oral gavage (p.o.) at 10 mg/kg and antiviral therapy with phillyrin (suspended in 0.4% carboxymethylcellulose sodium) was intraperitoneally injected (i.p.) at 15 mg/kg daily, respectively, starting 4 h after the PR8 challenge and continuing daily throughout the experiments. PBS was used as a gavage control (CON). The body weight of each mouse was observed and measured. At necropsy, 6 mice from each treatment group were designated to be sacrificed on the seventh day after the PR8 challenge. The lung was harvested and weighed to measure the lung index (lung weight/body weight). Then, it was divided into three portions for histopathology, quantitative real-time PCR (qRT-PCR) and Western blotting.

### Hematoxylin-eosin (H&E) staining

The removed lung tissue block was placed in a pre-prepared fixative (4% paraformaldehyde) for protein denaturation and coagulation in both cells and tissue. Varying alcohol concentrations were employed to dehydrate the tissue mass. The transparent tissues were embedded and made into paraffin blocks. The block was sectioned into thin slices after being embedded and fixed on the microtome. H&E was employed for cell staining according to the operating procedures.

### Cytokines assay in bronchoalveolar lavage fluid

The lungs were lavaged four times with PBS (0.5 mL). Following centrifugation (1000 rpm, 10 min, 4 ˚C), the supernatant was harvested to obtain BALF. The cytokines in BALF were determined with BioPlex-Pro-Mouse-Cytokine-23-plex-Assay-Kit (Bio-Rad, CA, USA) by following the kit’s protocols.

### RNA sequencing analysis

The RNAprep-Pure-Tissue-Kit (Tiangen Biotech, Beijing, China) was employed for the isolation of total RNA from the mouse lung tissue homogenate. The NanoPhotometer ® spectrophotometer and Agilent RNA 6000 Nano Kit (Agilent Technologies, CA, USA) were employed to detect the purity, concentration and integrity of RNA samples (OD260/280 = 1.8 ~ 2.0, OD260/230 = 2.0 ~ 2.4, RIN > 6, RNA amount > 1 µg). The qualified total RNA was enriched and purified with Oligo (dT) magnetic beads, and cDNA synthesis was carried out to construct cDNA libraries. The effective concentrations of the libraries (> 10 nM) were accurately quantified, the qualified libraries were sequenced with Illumina platform, and the raw reads were screened to acquire clean data. After alignment to the reference genome, the number of reads on each gene was counted, and the gene expression was calculated using the FPKM method. The samples were analyzed for differential expression, and the genes | log_2_Ratio | ≥ 1 and q < 0.05 were identified as differentially expressed genes (DEGs). Finally, KEGG database was used for further analysis of DEGs.

### Molecular docking

The Protein Data Bank (PDB) protein structure file for CXCR2 (PDB ID: 6LFL) was downloaded from the PDB database, and PyMOL software was used to extract the original ligand. The MOL2 structure file of phillyrin (MOL ID: MOL003305) was downloaded from the TCM Systems Pharmacology Database and Analysis Platform. Autodock software was utilized to hydrogenate CXCR2 and phillyrin, assign charges, and combine non-polar hydrogens. The active pocket was defined with the original ligand as the center, and the optimal candidate conformation was set. Lastly, Autodock Vina software was employed to realize the molecular docking of CXCR2 with phillyrin. The optimal binding energies between CXCR2 and phillyrin were calculated by Autodock vina software. The binding energy below − 5 kcal mol^− 1^ indicates that the target protein has good binding ability with phillyrin.

### Surface plasmon resonance (SPR) assay

Interaction between phillyrin and CXCR2 was analyzed using Biacore T200 system (GE Healthcare). The CXCR2 protein (MedChemExpress, USA) with a his-Tag was fixed on a carboxymethylated dextran chips pre-immobilized with NTA (GE Healthcare) via capture-coupling. The running buffer used in the experiments was adapted from PBS containing 5% DMSO and 0.05% Surfactant P20. Immobilization of CXCR2 was performed using a standard nickel chelation procedure. First, after extensive washing with 0.35 M EDTA (regeneration buffer) followed by running buffer without EDTA (eluent buffer), the second flow cell was loaded with 0.5 mM nickel solution (10 µL) to saturate the NTA surface with Ni2^+^ ions. Second, the running buffer with 3 mM EDTA was used to wash away Ni^2+^ ions which don’t stick firmly. Afterwards, 10 µL of His-tag CXCR2 (20 µg/ml) was injected into second flow cell, but not into the reference cell (first flow cell). To validate the affinity and kinetics of CXCR2 and phillyrin, phillyrin solutions of different concentrations ranging from 50 to 6.25 µM in a two-fold dilution step using running buffer was injected at a flow rate of 30 µL/min for 180 s with a dissociation time of 200 s, respectively, using multiple cycle kinetics. The chip was then regenerated with 2 M Glycine-HCl (pH 2.0). The binding constant was obtained using a steady state affinity model via a BIAcore evaluation software program.

### mRNA extraction and qRT-PCR

The RNAprep-Pure-Tissue-Kit (Tiangen Biotech) was utilized for isolation of total RNA from the mouse lung tissue homogenate and cell lysates. cDNA synthesis was conducted with FastKing-RT-Kit (Tiangen Biotech). Quantification of all gene expression was performed using the SuperReal PreMix Plus SYBR Green (Tiangen Biotech) on a Quant Studio^TM5^ Real-Time PCR instrument. The primer sequences are listed in Table S1. GAPDH was employed as the reference gene. Gene expression was measured using the $2^{\Delta \Delta {\rm CT}}$ method.

### Western blotting

Following tissue grinding, the protein was lysed in RIPA buffer. The protein content was examined using a BCA assay kit. After separation through SDS-PAGE, the protein was transferred onto PVDF membranes. The membranes were exposed to 5% milk for 3 h, and then placed in a refrigerator overnight at 4 °C with primary antibodies against β-actin (1:1000, Cell-Signaling-Technology, MA, USA), influenza A H1N1 hemagglutinin (1:1000, Abcam, Cambridge, UK), NLRP3 (1:1000, Cell-Signaling-Technology, MA, USA), ASC (1:1000, Cell-Signaling-Technology), Caspase 1 (1:1000, Cell-Signaling-Technology, MA, USA), and cleaved Caspase 1 (1:1000, Cell-Signaling-Technology). On the next day, the membranes were exposed to HRP–conjugated secondary antibody for 2 h at 4℃. Subsequently, the PVDF membranes were treated with a chemiluminescent substrate and then photographed. β-actin was employed as the reference protein. The relative values of the target proteins were calculated with ImageJ software.

### Statistical analysis

GraphPad Prism v8.0.2 was utilized for data and graph processing. Experimental data are expressed as means ± standard deviation (SD). Differences were compared with CON values by one-way ANOVA. P < 0.05 was deemed statistically significant.

## Results

### Phillyrin treatment alleviates lung inflammation caused by IAV

To investigate the effect of phillyrin treatment on IAV infection-stimulated pneumonia, BALB/c mice were intranasally infected with PR8 viral solution with LD_50_ concentration to establish a mouse model of IAV infection. Oseltamivir phosphate and phillyrin were administered starting 4 h after the PR8 challenge and continuing daily for 6 days. On 7 dpi, body weight, lung index and lung pathological changes were evaluated for each group of mice.

**Fig. 1:**
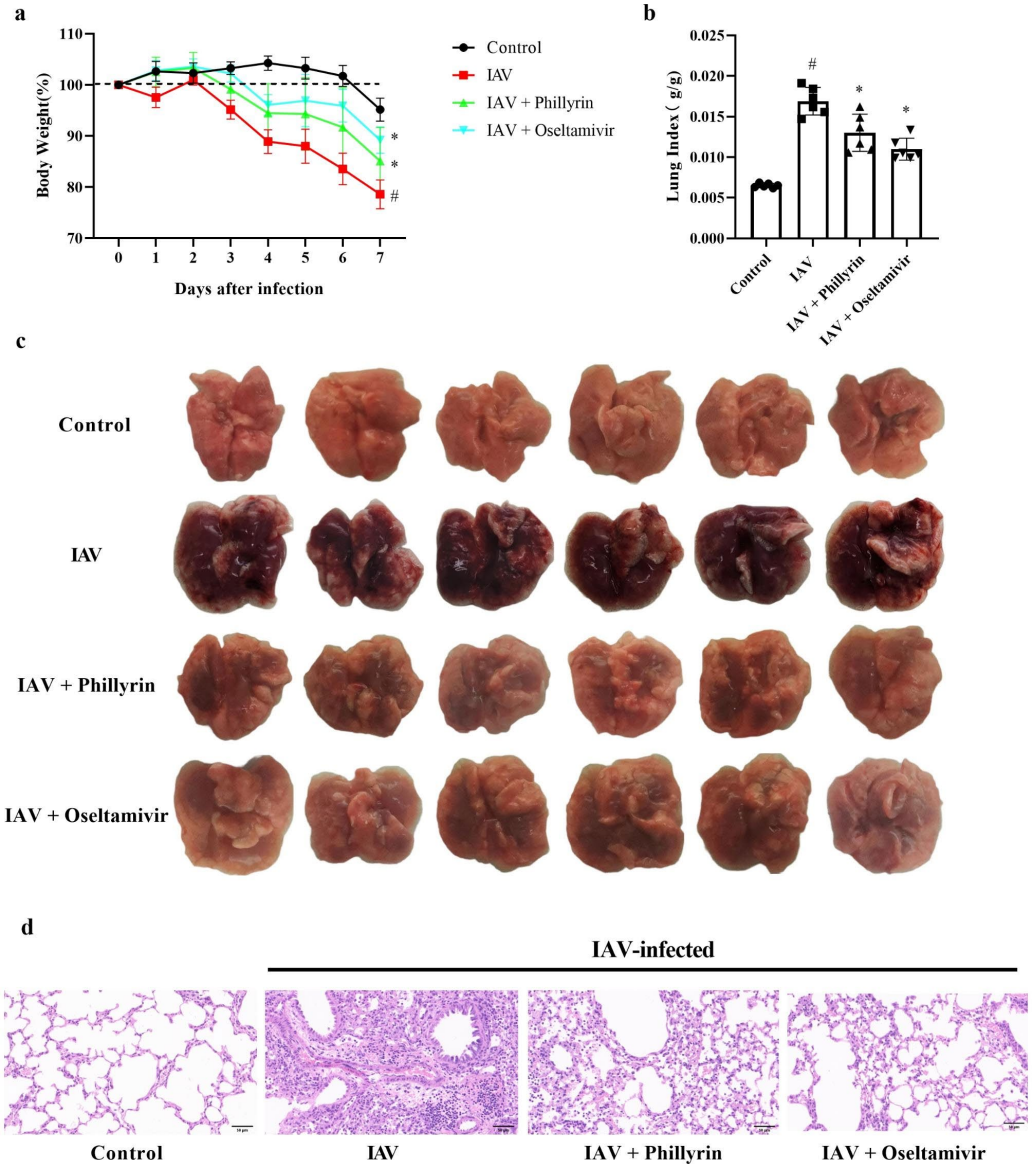
Phillyrin treatment alleviates lung inflammation caused by IAV. Percentage weight loss (**a**) and lung index (**b**) of mice on 7 dpi. Pulmonary lesions (**c**) and lung tissue pathological section (**d**) of mice in each group on 7 dpi. Original magnification: ×200; scale bar = 50 μm. Mean ± SD (n = 6/group). One-way ANOVA, # p < 0.05 vs. CON, * p < 0.05 vs. IAV.

The body weights of mice in different treatment groups are demonstrated in Fig. 1a. Infection of PR8 significantly reduced the body weights of virus-infected mice compared to the CON mice, while treatment with phillyrin (15 mg/kg) significantly alleviated this loss. Oseltamivir phosphate (10 mg/kg) was employed as a positive control. Macroscopic analysis revealed no significant pulmonary alterations in the CON group, but the lungs of virus-infected mice showed a large congestion area at 7 dpi. Furthermore, the lung index of virus group was higher than that of CON group, but phillyrin ameliorated pulmonary congestion and reduced the lung index, suggesting its beneficial effect on pulmonary inflammation (Fig. 1b-c. H&E staining indicated that no pathological alterations occurred in the lungs of CON group. The lesions of virally infected mice included inflammatory cell infiltration around blood vessels and bronchioles. Of note, the above-mentioned inflammatory features were alleviated after treatment with phillyrin, confirming the relief of microscopic inflammation by phillyrin (Fig. 1d).

### Phillyrin suppressed influenza viral replication and reduced influenza virus-induced CPE in vitro

Multiplication of the influenza virus may require metabolites, such as amino acids and nucleotides, together with cellular machinery for the synthesis of genes and proteins, thus generating viral particles. IAV contains 8 negative-sense RNA segments encoding 11 major proteins. Among them, the glycoprotein hemagglutinin (HA) is encoded by the HA segment, which can regulate virus entry into a host via binding with sialic acid in the host cell receptors. The matrix segment encodes the matrix protein M1, which is responsible for the budding process and the ion channel M2 protein. Nucleoprotein (NP) is the skeleton structure of influenza viral ribonucleoprotein (vRNP) complexes, are all transported into the nucleus where viral replication and transcription occur [12, 13].

**Fig. 2:**
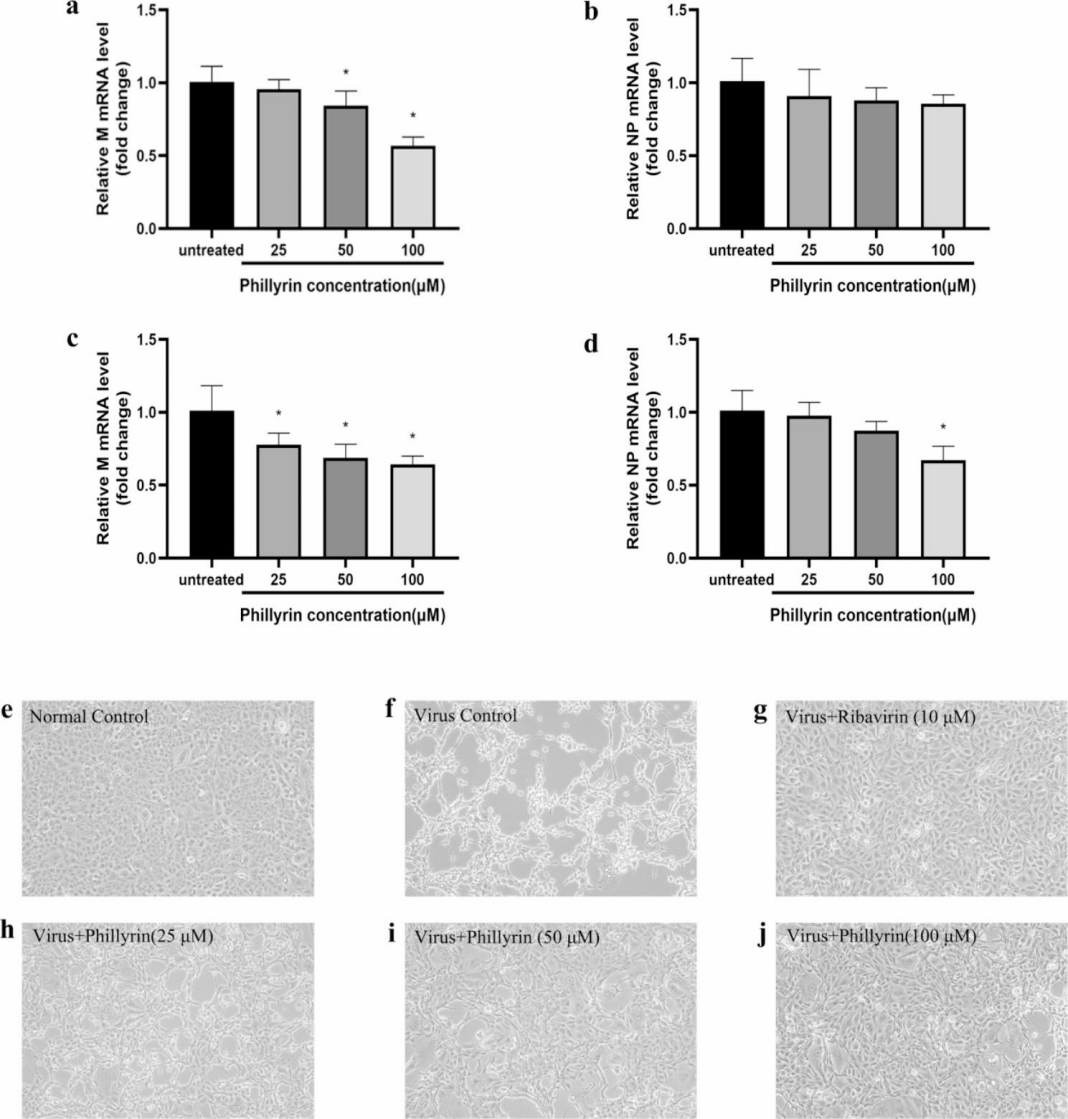
Phillyrin suppressed influenza viral replication and reduced influenza virus-induced cytopathic effect (CPE). Madin Darby Canine Kidney (MDCK) cells were inoculated with IAV (PR8) at multiplicity of infection (MOI) 0.005. After 2 h adsorption, virus inoculum was removed and replaced with freshly prepared infectious media with phillyrin at different concentrations (100, 50, 25µM) or ribavirin at 10µM (as a positive control). Viral RNA (M mRNA and NP mRNA ) replication (**a-d**): Cells were collected at12hpi and 24hpi respectively, and RNA was isolated, and M mRNA and NP mRNA were determined using quantitative real-time polymerase chain reaction (qRT-PCR). % of M mRNA levels (**a** and **c**) and NP mRNA levels (**b** and **d**) in virus-infected samples in the presence of Phillyrin relative to virus infection alone was calculated. The graphs represent the mean and standard deviation of three independent experiments of treatment by Phillyrin. Significant differences were identified by one-way analysis of variance (ANOVA), *p < 0.05, relative to virus infection alone without treated group. Virus-induced CPE was recorded under microscopy at 48hpi (**e-j**). Results shown are from a typical experiment.“Normal Control”is the negative control with no influenza virus, cultured for the same time period in the presence of phillyrin and ribavirin

To determine the effect of phillyrin on influenza viral replication, the relative levels of M and NP mRNA expression were evaluated by Real-time RT-PCR assay at 12 h hpi and 24hpi, respectively. As shown in Fig. 2a, after treatment with phillyrin (50, 100µM) for 12 h, the IAV M mRNA levels decreased to about 84% and 57% of that of untreated cells after phillyrin treatment, respectively. When the duration of treatment with phillyrin extended to 24 h, the results showed more pronounced suppressive effect of phillyrin (Fig. 2c) on M mRNA levels in a dose-dependent manner. The expression of the NP gene was highest in the untreated group and lower in all other treated groups with phillyrin, however, the difference did not reach statistical significance in any of group at 12hpi (Fig. 2b). The IAV NP mRNA levels decreased to about 67% of that of untreated cells only after phillyrin (100µM) treatment and decrease was significant at 24hpi (Fig. 2d). Moreover, virus-induced CPE was recorded under microscopy at 36 hpi. Direct microscopic observation at 36 hpi (Fig. 2f-j) showed that treatment with phillyrinresulted in significant reduction of virus-induced CPE by IAV compared with untreated mock control. Ribavirin was employed as a positive control. These results demonstrate that phillyrin has potent antiviral effects against influenza infection in cell culture. Their antiviral effects are demonstrated through reduction of influenza virus-induced CPE as well as suppression of viral genome RNA replication.

In vivo, IAV infection obviously increased the mRNA level of M (Fig. S1a) and the protein level of HA (Fig. S1b) in the lungs at 7 dpi. However, phillyrin cannot rescue this IAV-induced increase of main genome and protein synthesis,which may be due to the diversity of cell composition characteristics of lung tissue and different single-cell environments.

### Phillyrin attenuates the secretion of multiple cytokines and chemokines in mouse BALF upon IAV Infection

**Fig. 3:**
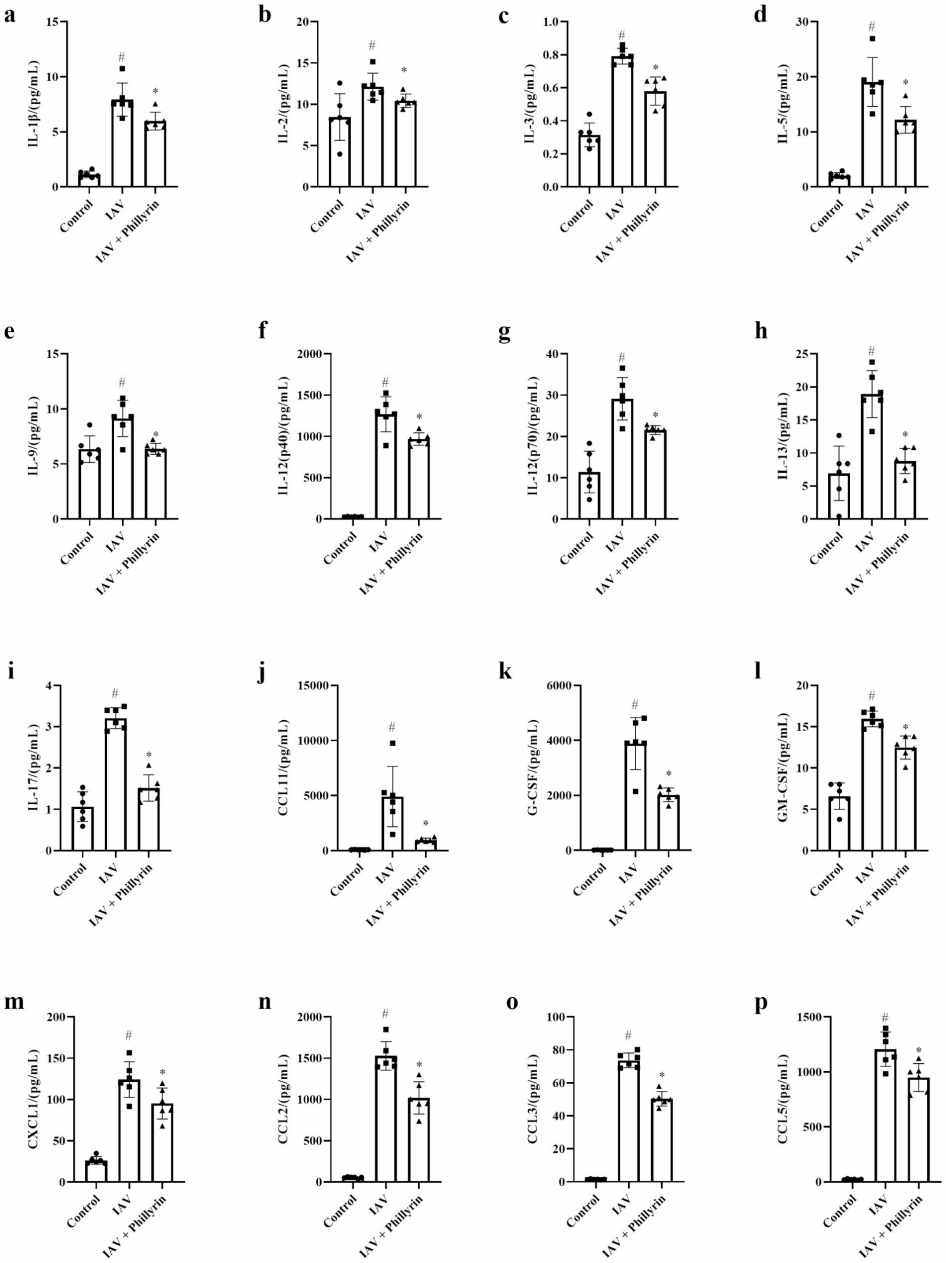
Phillyrin attenuates the secretion of multiple cytokines and chemokines in mouse BALF upon IAV infection. On 7 dpi, the levels of 23 cytokines in BALF of different treatment groups were detected. The figure shows the cytokines with significant changes in secretion level after phillyrin treatment. Mean ± SD (n = 6/group). One-way ANOVA, # p < 0.05 vs. CON, * p < 0.05 vs. IAV.

To assess the effect of phillyrin on mouse BALF, the secretion of a total of 23 cytokines and chemokines associated with IAV-induced lung inflammation was investigated. Notably, the levels of these cytokines and chemokines were remarkably higher in IAV-infected group compared to CON group. However, treatment with phillyrin dramatically attenuated the production of 16 pro-inflammatory cytokines and chemokines, namely, IL-1β, IL-2, IL-3, IL-5, IL-9, IL-12 (p40), IL-12 (p70), IL-13, IL-17, GM-CSF, G-CSF, CCL11, CXCL1, CCL3, CCL4 and CCL5 (Fig. 3). The other results were shown in Fig. S2.

### The cytokine-cytokine receptor interaction is involved in the suppressive effect of phillyrin in IAV-induced Pneumonia

**Fig. 4:**
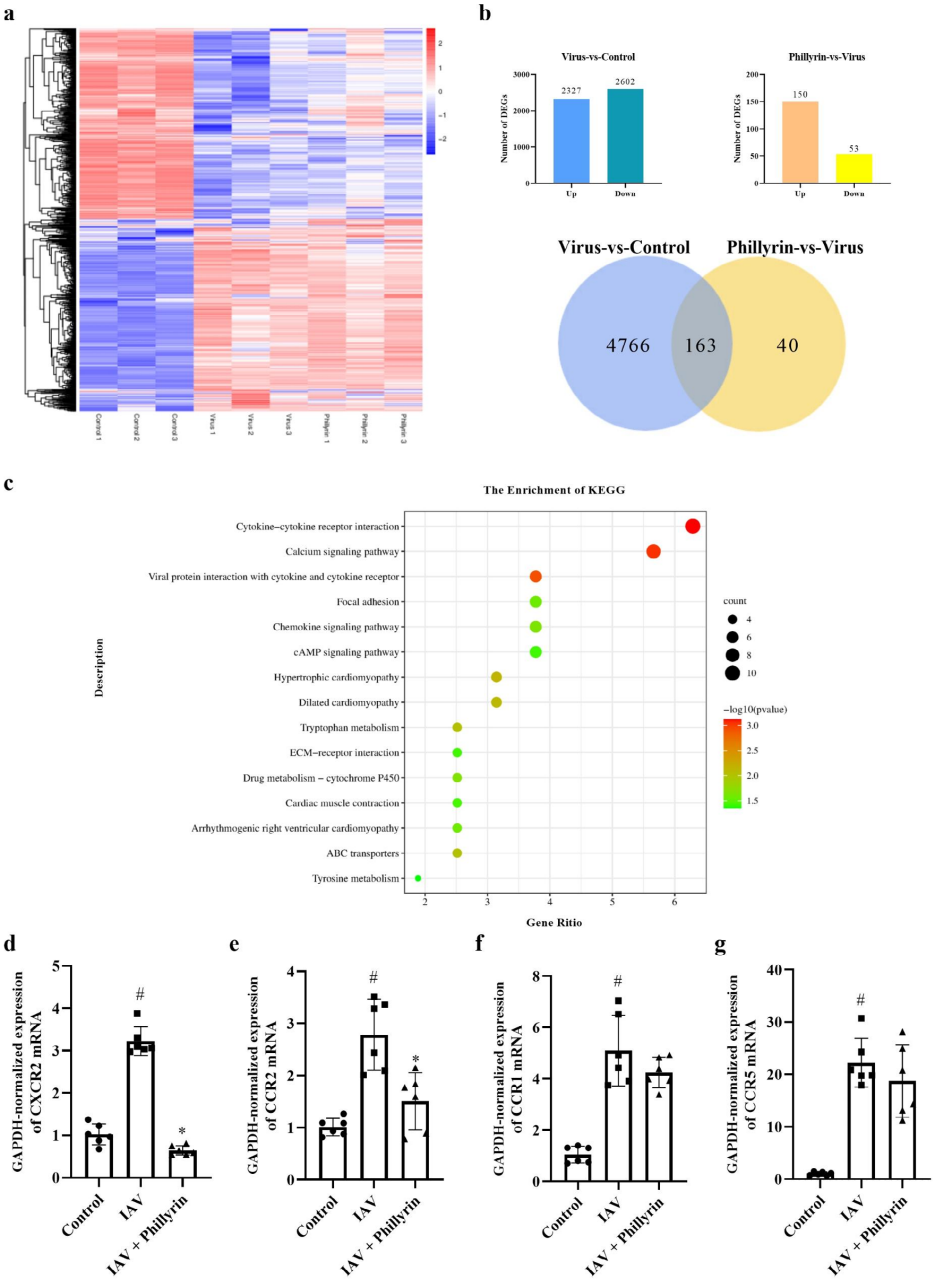
The cytokine-cytokine receptor interaction is responsible for the suppressive effect of phillyrin on IAV-induced pneumonia. (**a**) Hierarchical cluster analysis of DEGs among CON group, virus group and phillyrin group. Red and blue hues indicate upregulated and downregulated expression, respectively. (**b**) The quantity of DEGs in virus-vs.-CON and phillyrin-vs.-virus groups, and comparison of DEGs between virus-vs.-CON and phillyrin-vs.-virus. (**c**) KEGG enrichment bubble diagram with common DEGs. The mRNA expression of CXCR2 (**d**), CCR2 (**e**), CCR1 (**f**), CCR5 (**g**). Mean ± SD (n = 6/group). One-way ANOVA, # p < 0.05 vs. CON, * p < 0.05 vs. IAV.

To further explore the underlying mechanisms of phillyrin in treating IAV-induced pneumonia, we extracted RNA from the lung tissues of each mouse and proceeded RNA-seq analysis. By comparing the gene expression of virus group and CON group, 4929 DEGs were identified and phillyrin treatment resulted in 203 DEGs (Fig. 4a and b). In brief, 163 overlapping DEGs were affected in the virus, CON and phillyrin groups (Fig. 4b). These DEGs might be the important therapeutic effector molecules of phillyrin treatment on IAV-induced pneumonia.

Furthermore, pathway enrichment analysis of the 163 DEGs was performed through the KEGG database. The results showed that 15 pathways were markedly enriched for these DEGs (P < 0.05; Table 1), including the calcium signaling pathway, cytokine-cytokine receptor interaction, viral protein interaction with cytokine and cytokine receptor, chemokine signaling pathway, focal adhesion and cAMP signaling pathway (Fig. 4c). It is worth noting that there are three classic chemokine signaling pathways (ko04060, ko04061, and ko04062). These results suggest that the cytokine-cytokine receptor interaction plays a crucial role in enhancing the therapeutic effect of phillyrin on IAV-induced pneumonia.

**Table 1:** KEGG pathway significantly enriched

Pathway	KEGG Class	Pathway Name	Count	Background Number	P Value
ko04060	Environmental Information Processing; Signaling molecules and interaction	Cytokine-cytokine receptor interaction	10	292	7.46 × 10^− 4^
ko04020	Environmental Information Processing; Signal transduction	Calcium signaling pathway	9	240	8.99 × 10^− 4^
ko04061	Environmental Information Processing; Signaling molecules and interaction	Viral protein interaction with cytokine and cytokine receptor	6	95	0.0012
ko05410	Human Diseases; Cardiovascular disease	Hypertrophic cardiomyopathy	5	91	0.0072
ko05414	Human Diseases; Cardiovascular disease	Dilated cardiomyopathy	5	94	0.008
ko00380	Metabolism; Amino acid metabolism	Tryptophan metabolism	4	52	0.0095
ko02010	Environmental Information Processing; Membrane transport	ABC transporters	4	52	0.0095
ko00982	Metabolism; Xenobiotics biodegradation and metabolism	Drug metabolism - cytochrome P450	4	71	0.0219
ko04062	Organismal Systems; Immune system	Chemokine signaling pathway	6	192	0.0228
ko05412	Human Diseases; Cardiovascular disease	Arrhythmogenic right ventricular cardiomyopathy	4	77	0.0271
ko04510	Cellular Processes; Cellular community - eukaryotes	Focal adhesion	6	201	0.0272
ko04260	Organismal Systems; Circulatory system	Cardiac muscle contraction	4	87	0.0370
ko04024	Environmental Information Processing; Signal transduction	cAMP signaling pathway	6	220	0.0380
ko04512	Environmental Information Processing; Signaling molecules and interaction	ECM-receptor interaction	4	88	0.0381
ko00350	Metabolism; Amino acid metabolism	Tyrosine metabolism	3	40	0.0446

Moreover, the levels of 5 chemokines such as CCL11/Eotaxin, CXCL1/KC, CCL5/RANTES, CCL2/MCP-1 and CCL3/MIP-1α were dramatically reduced by phillyrin in BALF of IAV-infected mice as mentioned previously (Fig. 3). These inflammatory mediators normally recruit cells of the innate immune system through selectively binding to the corresponding receptors. CCL11 is mainly involved in the recruitment of eosinophils into the lung, and its receptor is CCR3 [14]. CCL3 and CCL2 play a role in inflammatory responses through binding to the receptors CCR1, CCR2 and CCR5 [15]. As one of the natural ligands of CCR5, CCL5/CCR5 can mediate the recruitment and activation of neutrophils and monocytes during influenza, and CXCL1 can chemotactic T cells, monocytes, neutrophils and other immune cells when combined with its specific CXCR2 receptor [16–18]. Previous findings showed that activation of cytokine receptors at the initial stage of influenza infection could ensure proper recruitment of white blood cells and activation of antiviral pathway in epithelial cells. However, continuous or excessive activation of cytokine receptors during severe influenza infection may aggravate inflammatory reaction, leading to increased lung injury [19, 20]. One possible strategy for enhancing anti-inflammatory effects is to selectively bind and neutralize the chemokines and chemokine receptors. In view of the important role of chemokine receptors, we further detected the mRNA expression levels of CCR3, CXCR2, CCR2, CCR1 and CCR5 in the lungs. The findings demonstrated that the mRNA levels of CCR5, CCR2, CCR1 and CXCR2 genes were increased after influenza infection. Phillyrin treatment could significantly reduce the expression of CCR2 and CXCR2, among which the latter was more significantly regulated by phillyrin (Fig. 4d-g). CCR3 gene was undetermined due to its low expression in the lungs (Results not listed).

### Phillyrin has good binding ability to CXCR2

**Fig. 5:**
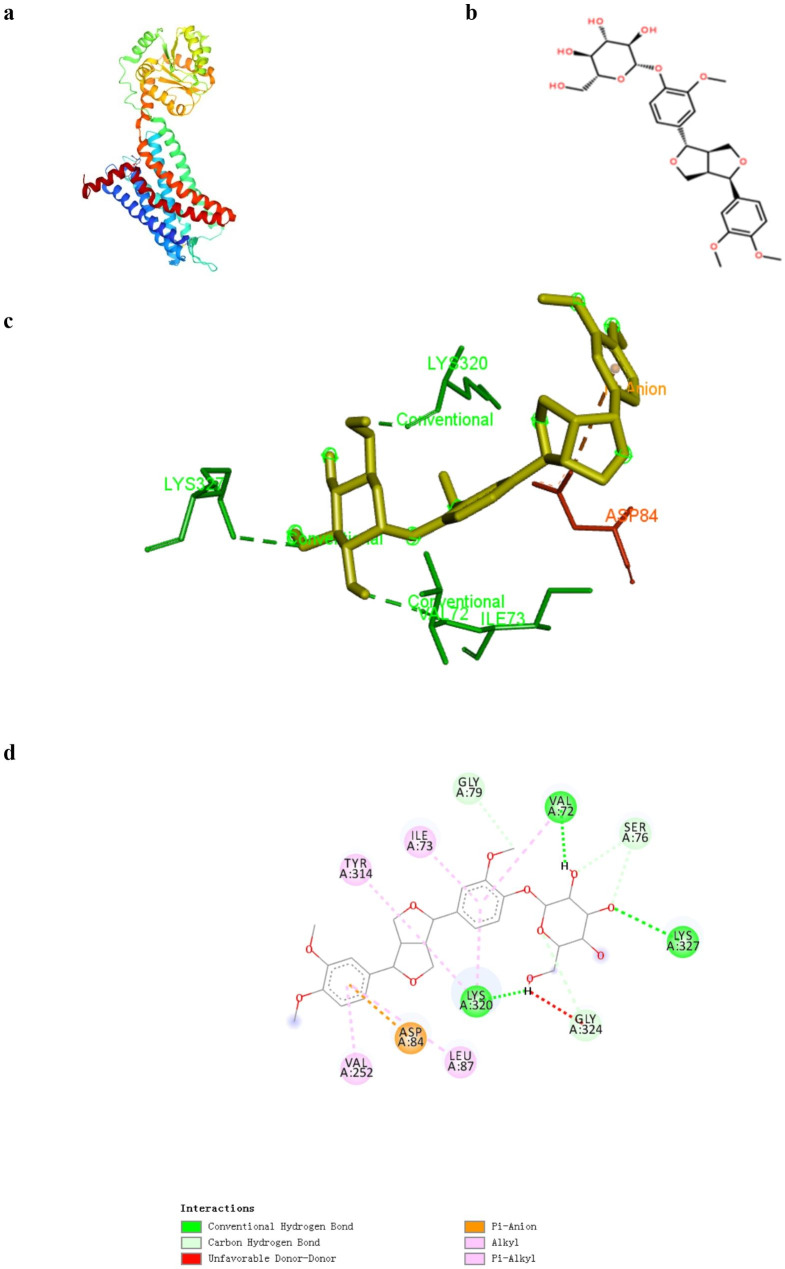
Phillyrin has good binding ability to CXCR2. (**a**) Protein structure of CXCR2. (**b**) Molecular structure of phillyrin. (**c**) 3D diagrams showed the distribution of hydrogen donors and receptors in active pockets. (**d**) 2D diagrams showed the bond types and amino acid residues. Green and orange dashed lines indicate hydrogen bonds and Pi-Anion, respectively

CXCR2 is a critical target in the efforts to suppress neutrophilic inflammation. Excessive neutrophil influx is related to greater disease severity in patients with influenza infection. A number of studies on the experimental inhibition of CXCR2 have reported its ameliorative effects on inflammatory responses and lung injury in the sublethal influenza murine models [21–23]. We verified the affinity of phillyrin to CXCR2 based on molecular docking. Generally, the molecular docking score of less than 0 kcal mol^− 1^ indicates that ligands can spontaneously bind to receptors, and the score less than − 5 kcal mol^− 1^ indicates that they have good binding ability [24, 25]. Our results showed that binding affinity was − 8.9 kcal mol^− 1^ between phillyrin and CXCR2, and the drug-target had good binding ability. In addition, their bonding types were hydrogen bonds (from residues LYS320, LYS327 and VAL72) and electrostatic force (Fig. 5.). As a special intermolecular force, hydrogen bond is a critical index to measure the affinity between protein and small molecules. Therefore, CXCR2 may be an important drug-target of phillyrin against IAV-induced pneumonia.

**Fig. 6:**
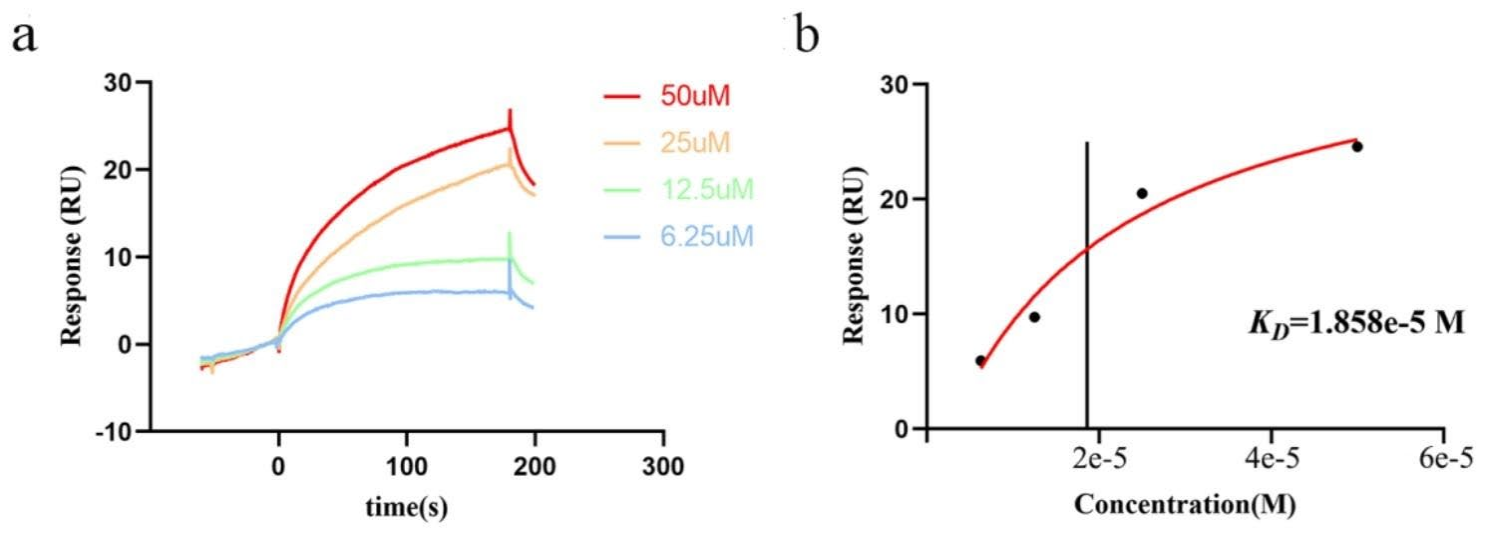
The binding affinity between phillyrin and CXCR2. Interactions of CXCR2 with phillyrin measured by SPR. The CXCR2 with a his-Tag was fixed on the NTA sensor chip via capture coupling and serial dilutions of phillyrin (6.25µM, 12.5µM,25µM,50µM) were used as analytes. Changes in plasmon resonance are shown as response units. (**a**) Multicycle kinetics using CXCR2 as ligand and phillyrin as analyte. Different colored lines correspond to different concentrations of Phillyrin; (b)The KD value of phillyrin and CXCR2 was calculated by non-linear regression analysis

To further confirm whether phillyrin is the direct binding to CXCR2. An SPR assay was then performed to evaluate the binding affinity of phillyrin and CXCR2. As shown in Fig. 6a, phillyrin bound to CXCR2 in a dose-dependent manner. The response units at equilibrium were plotted against phillyrin concentrations (Fig. 6b), and the binding affinity constant (KD) value was calculated by non-linear regression. The results showed that phillyrin bound to CXCR2 with a KD value of 18.58µM, indicating that phillyrin presented strong affinity for CXCR2.

### Phillyrin inhibits overactivation of NLRP3 inflammasome

**Fig. 7:**
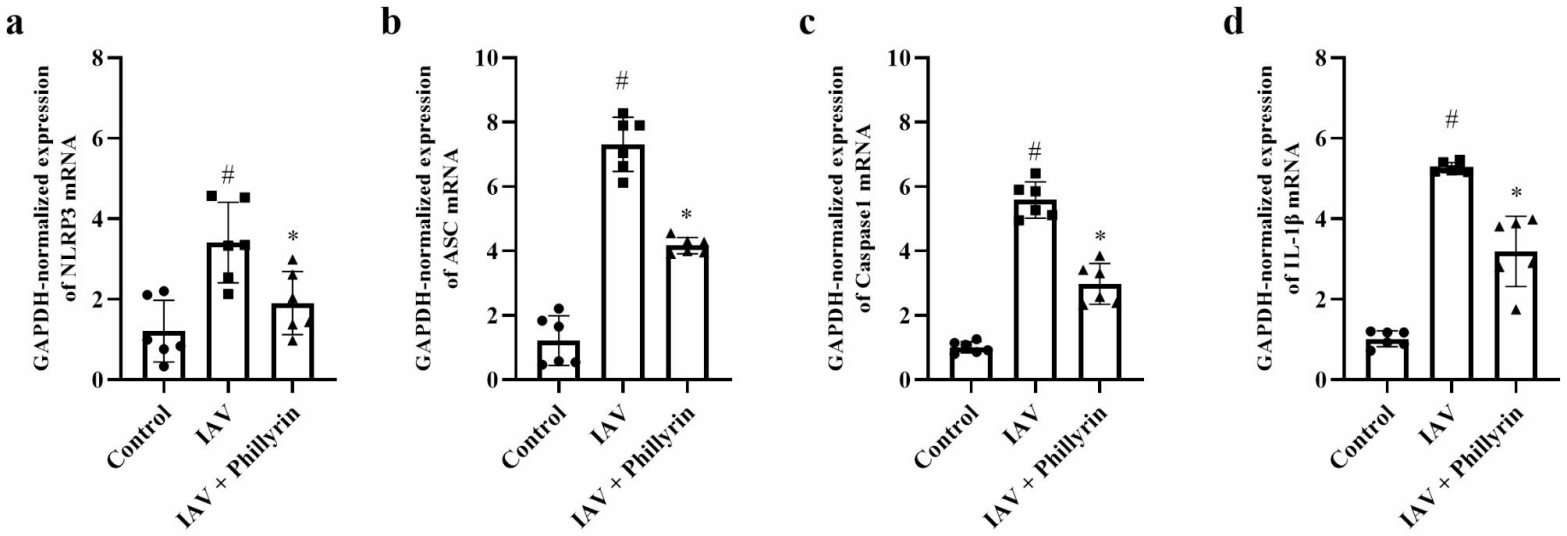
Phillyrin inhibits mRNA expression of NLRP3 inflammasome. The mRNA expression of NLRP3 (**a**), ASC (**b**), Caspase 1 (**c**), and IL-1β (**d**) in the lungs on 7 dpi, as detected by qRT-PCR. Mean ± SD (n = 6/group). One-way ANOVA, # p < 0.05 vs. CON, * p < 0.05 vs. IAV.

**Fig. 8:**
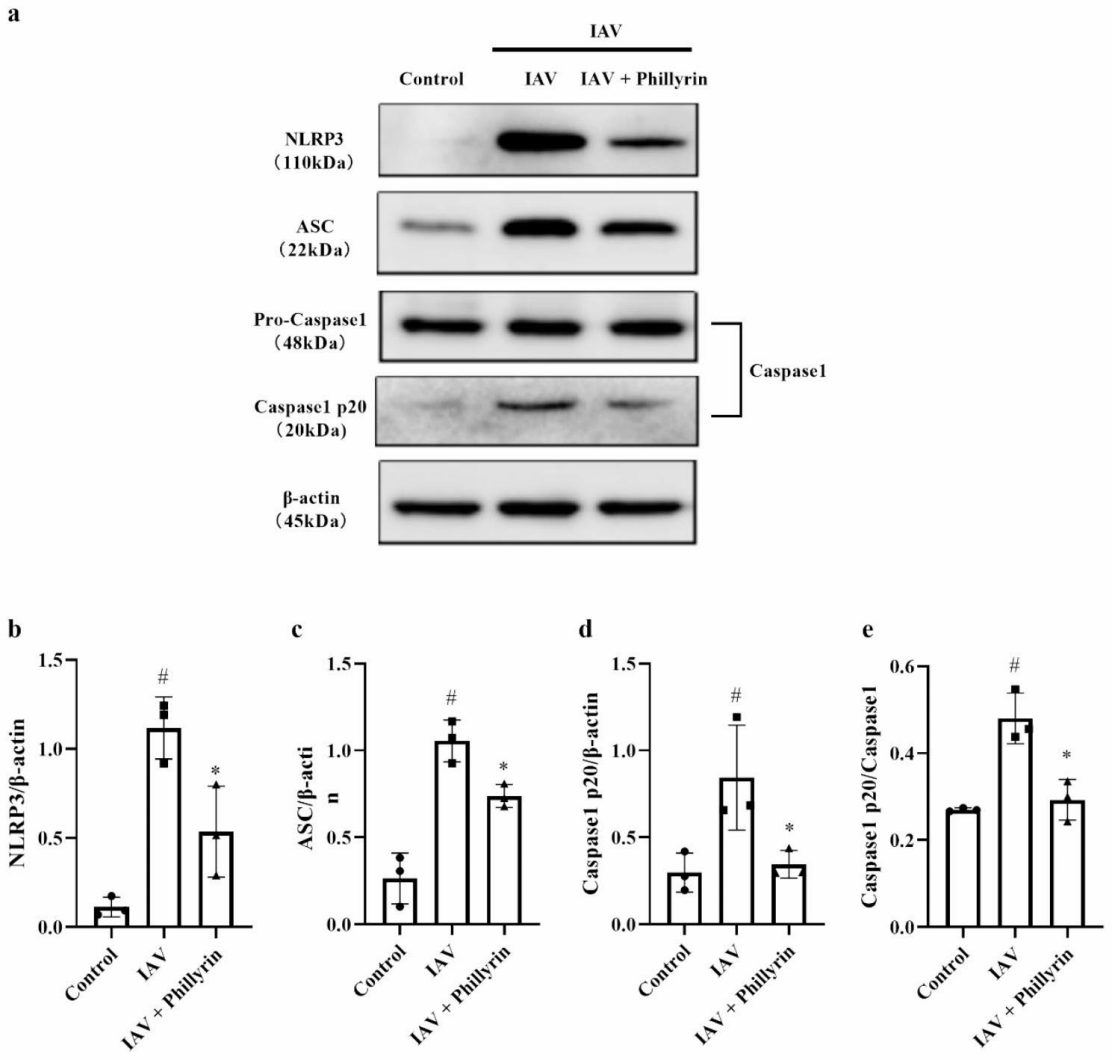
Phillyrin inhibits protein expression of NLRP3 inflammasome. The protein levels of NLRP3 (**b**), ASC (**c**) and Caspase 1 (**d**), and Caspase1 p20 subunit/Caspase1 (**e**) in the lungs on 7 dpi were detected by Western blotting. Mean ± SD(n = 3 independent experiments) One-way ANOVA, # p < 0.05 vs. CON, * p < 0.05 vs. IAV.

Previous studies have reported that CXCL1-CXCR2 axis could regulate NLRP3 inflammasome activation in some inflammatory scenarios [26]. IAV can induce excessive secretion of cytokines by triggering NLRP3 activation, thus resulting in lung damage and death [27, 28]. Specifically, hyperinflammatory responses, such as the secretion of NLRP3-dependent IL-1β, are characteristic features of severe IAV infection [29, 30]. In our study, phillyrin downregulated IL-1β production and inflammatory responses in BALF from H1N1-induced pneumonia mice (Fig. 3a). To further elucidate the anti-inflammatory mechanisms of phillyrin, the expression levels of NLRP3 inflammasomes and the Caspase1 p20 subunit (representing activated Caspase1)/Caspase1 were analyzed. Notably, the mRNA and protein expression levels of Caspase1, ASC and NLRP3 were all elevated in the lungs of mice with H1N1-induced pneumonia, but these trends were suppressed in the phillyrin group (Figs. 7a-c and 8a-d). Besides, in the virus group, the proportion of Caspase1 p20 to Caspase1 and the mRNA expression of IL-1β were higher than those in CON group, but were obviously reduced in phillyrin-treated mice (Figs. 7d and 8a and e). These findings suggest that phillyrin can suppress the overactivation of NLRP3 inflammasomes in mice with IAV-induced pneumonia.

### Phillyrin has the similar effect as CXCR2 antagonist

**Fig. 9:**
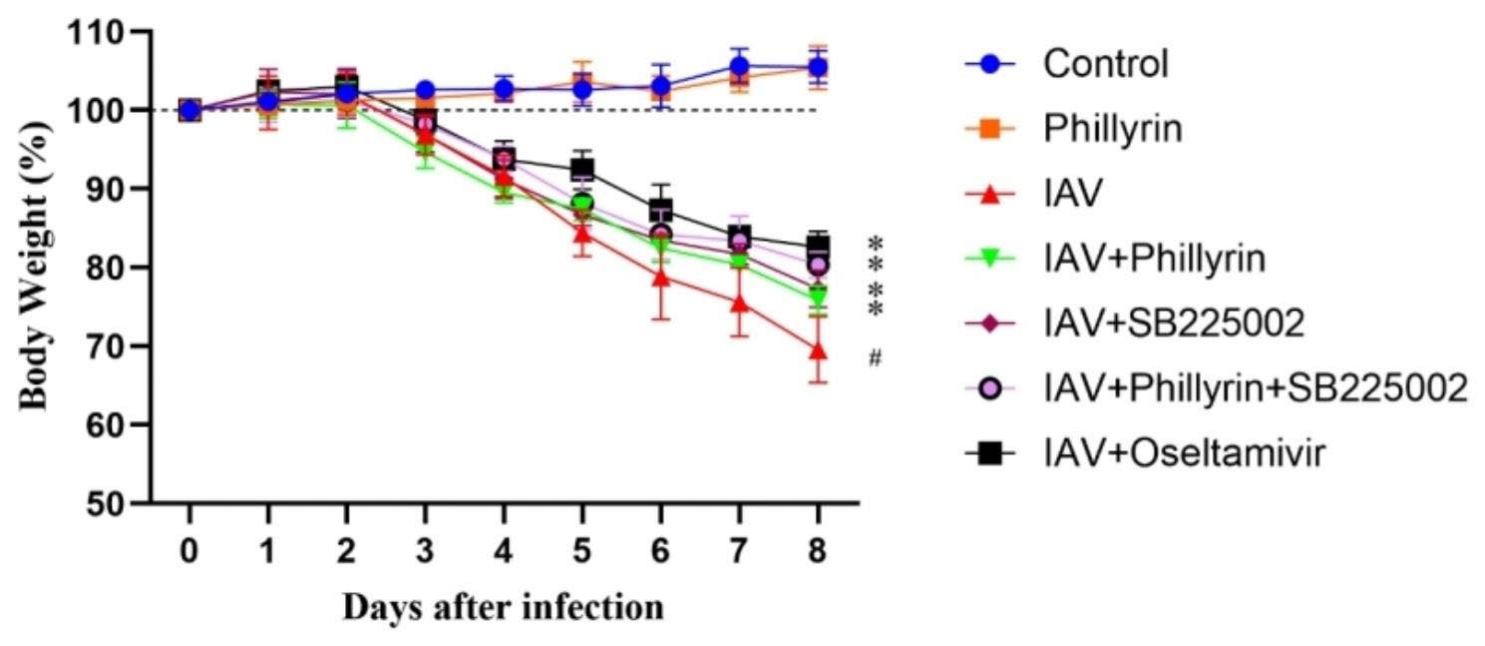
Phillyrin and CXCR2 antagonist SB225002 treatment alleviates percentage weight loss caused by IAV. Briefly, BALB/c mice were randomized into several groups: Control group (n = 6, Saline-treated), Phillyrin group (n = 6, intraperitoneal injection (i.p.) of 15 mg/kg Phillyrin once a day from 0 day to 4 days without infection ), IAV group (n = 6, intranasally challenged with PR8), IAV + Phillyrin group (n = 6, i.p. of 15 mg/kg Phillyrin once a day from 0 day to 4 days postinfection ), IAV + SB225002 group (n = 6, i.p. of 20 μm SB225002 once a day from 0 day to 4 days postinfection ), IAV + Phillyrin + SB225002 group (n = 6, daily i.p. of 20 μm SB225002 from 1 days pre- to 4 days post-infection and i.p. of 15 mg/kg Phillyrin once a day from 0 day to 4 days postinfection), IAV + oseltamivir group (n = 6, orally administrated with 10 mg/kg oseltamivir once a day from 0 day to 4 days postinfection). Phillyrin and SB225002 was suspended in 0.4% carboxymethylcellulose sodium. The body weight change of each group were observed daily following PR8 challenge for a total of 8 days. Mean ± SD (n = 6/group). One-way ANOVA, # p < 0.05 vs. Control, * p < 0.05 vs. IAV.

I repeated an in vivo experiment in the context of CXCR2 antagonist SB225002 (a potent, selective and non-peptide CXCR2 antagonist, MedChemExpress). The body weights of mice in different treatment groups are demonstrated in Fig. 9. The result showed that infection of PR8 significantly reduced the body weights of virus-infected mice compared to the control mice, while treatment with phillyrin-only, CXCR2 antagonist SB225002-only, phillyrin and SB225002 synergistically significantly alleviated this loss. Oseltamivir phosphate was employed as a positive control. Compared to phillyrin-only group or SB225002-only group, we found that phillyrin and SB225002 synergistically enhanced weight regain. This data indicated that phillyrin has the similar effect as CXCR2 antagonist in improving virus-induced weight loss. However, further testing is needed to determine what changes have occurred in the CXCR2-NLRP3-inflammasome pathway.

## Discussion

The influenza virus causes seasonal influenza and can even lead to outbreak and epidemic worldwide [3, 31]. Viral pneumonia from IAV infection is a common complication and leading cause of death [32]. As a lack of drugs used to control viral acute lung injury continues to persist, the development of new therapeutic agents is essential. This study found that phillyrin, a monomer TCM ingredient with anti-inflammatory activity, exerted therapeutic effects on the pneumonia induced by IAV infection. The results showed that phillyrin treatment alleviated pneumonia induced by virus infection and significantly ameliorated the upregulation of multiple pro-inflammatory cytokines and chemokines (e.g., IL-1β, CXCL1, CCL3, CCL2, CCL5 and so on) in BALF. More importantly, the results of RNA sequencing analysis revealed that the cytokine-cytokine receptor interaction plays an essential role in regulating the therapeutic effect of phillyrin on influenza-induced pneumonia, and the mRNA expression level of CXCR2 was confirmed to be markedly inhibited by phillyrin. We performed computerized molecular docking of phillyrin with CXCR2 and verified good binding activity. Furthermore, SPR analysis, the gold standard method for studying protein-protein interactions, confirmed that phillyrin direct bound to CXCR2 with high affinity activity. All of these results indicate that CXCR2 is a potential therapeutic target for phillyrin. We also showed that phillyrin treatment could affect the mRNA and protein expression levels of Caspase1, ASC and NLRP3 in the lung homogenates of mice infected with H1N1. The ameliorative effect of phillyrin on influenza-induced pulmonary pathological damage may be partly related to the antagonization of CXCR2 and inhibition of NLRP3 inflammasome activation.

Influenza patients are often afflicted with severe pneumonia that is characterized by excessive infiltration of leukocytes and secretion of proinflammatory cytokines [33, 34]. Despite the inflammatory response produced by the immune host to remove the virus, the elevated production of different inflammatory cytokines typically contributes to the pathogenesis of IAV-induced acute lung injury [35–37]. In the present study, phillyrin (15 mg/kg) remarkably decreased the lung index, relieved the extensive degeneration and necrosis of the bronchial and bronchiolar epithelium after IAV infection and inhibited the accumulation of multiple pro-inflammatory cytokines and chemokines, revealing that phillyrin can alleviate pulmonary inflammation in mice with virus-induced pneumonia.

- Our study also found that oseltamivir, as a positive control, led to similar reductions in pathology, suggesting that a decrease in viral replication alone can alleviate the pathological and inflammatory responses.To determine the effect of phillyrin on influenza viral replication, the relative levels of M and NP mRNA expression and virus-induced CPE at indicated time points in vitro as well as the contents of M mRNA and the levels of HA protein of lungs at 7 dpi i*n vivo* were examined. The cell experiments verified the inhibitory effect of phillyrin on influenza viral multiplication and the results were similar to the previous report [38], however, the inhibitory trend was not significant i*n vivo.* Unlike a single cellular environment, lung tissue is composed of a variety of cells such as respiratory epithelial cells and macrophages [39]. The higher levels of viral genomic replication, transcription, and translation in alveolar epithelial cells probably makes IAV maintain a certain amount in lung tissue to partially “counteract” the inhibition of phillyrin on viral multiplication. Therefore, in the present study, although the research in vitro have confirmed the inhibitory effect of phillyrin on influenza viral multiplication, this inhibition was not significant i*n vivo*. The amelioration of phillyrin on pulmonary inflammation in influenza viral pneumonia mice is not mainly dependent on its antiviral activity.

The pathogenesis of pneumonia caused by influenza is not only directly associated with viral cytopathology but also forms an overzealous host immune response. Neutrophils have been recognized as an important cell type that leads to deterioration in lung functions [40]. When the virus reaches the lung epithelium, neutrophils are recruited by the immune/non-immune cells, leading to the production of chemokines (e.g., CXCL1/CXCL2) in mice [1, 41]. These chemokines act through their receptor CXCR2 in different types of cells. Previous studies have shown that the chemokine receptor CXCR2 has an essential role in regulating pulmonary inflammation and damage during severe IAV infection. The downregulation of CXCR2 could prevent such aggravated response, even after sequential IAV infections, without affecting the virus-specific adaptive immune responses [22]. In the present work, DEGs and potential pathways in the lung tissue of the virus group and phillyrin group were evaluated using RNA-seq and qRT-PCR. The data suggest that the cytokine-cytokine receptor interaction plays a vital role in regulating the inhibitory effect of phillyrin on IAV-induced pneumonia. It was found that the expression of CXCL1 and CXCR2 was markedly upregulated in the BALF and lungs of mice with IAV infection. Interestingly, phillyrin treatment could remarkably decrease the expression of CXCR2 and the secretion of CXCL1. Furthermore, we performed the computerized molecular docking of phillyrin with CXCR2 and confirmed good binding activity between them, indicating that phillyrin is a potential CXCR2 inhibitor.

The CXCL1-CXCR2 axis has been reported to be involved in NLRP3 inflammasome activation and functions in some inflammatory scenarios. Tang and co-workers showed that this axis might induce inflammatory response in diabetic nephropathy by promoting NLRP3 inflammasome overactivation [26]. The activation of NLRP3 inflammasomes in LPS-primed macrophages was observed to be specific for CXCL1 and CXCL2 but not other chemokines such as CCL2 and CCL5 [42–44]. The inflammatory response is an integral part of IAV infection. Recently, the NLRP3 inflammasome complex has been revealed to have essential roles in both detrimental and protective immune responses during IAV infection. NLRP3 inflammasomes are oligomeric signaling platforms that induce Caspase1-associated pyroptotic cell death and mature the prototypic inflammatory cytokines IL-18 and IL-1β [45]. Recent studies have highlighted that inhibition of NLRP3 inflammasome activation can effectively treat influenza viral pulmonary inflammation. MCC950, an inhibitor specific to NLRP3 inflammasome, has been shown to improve the survival and alleviate pulmonary inflammation in IAV-infected mice by downregulating the expression of Caspase1 and NLRP3 [46]. In this study, phillyrin downregulated the expression levels of Caspase1, ASC and NLRP3 in the lungs of mice with virus-induced pneumonia and reduced the protein ratio of Caspase1p20/Caspase1, thereby inhibiting NLRP3 inflammasome overactivation. However, the previous report [42] shows that ILK and PKCµare the linkage to CXCL1 or 2/CXCR2 and NLRP3 inflammasome activation. The detection of phosphor-ILK and phosphor-PKCµ level will help to further clarify if phillyrin affect inflammasome activity upon influenza A virus infection in the furture.

## Conclusion

This study demonstrates that phillyrin can ameliorate pulmonary inflammation in mice with virus-induced pneumonia, and its anti-inflammatory mechanisms may be through antagonizing CXCR2 and suppressing NLRP3 inflammasome overactivation in the lung of infected mice. Although we observed this phenomenon and the possible mechanism, lung tissue–specific CXCR2 knockout mouse models should be constructed in future experiments in order to evaluate this pathway in vivo. In addition, some key questions also remain to be resolved. For instance, is it specific for CXCL1 or CXCR2 that phillyrin inhibits NLRP3 inflammasome activation in the lungs of IAV-infected mice? If so, what is the precise molecular mechanism by which phillyrin ameliorates influenza viral pneumonia through depressing NLRP3 inflammasome activation via declining CXCR2 expression? Still, although significant work remains to be conducted, our findings provide novel insights into the treatment of IAV-induced pulmonary inflammation.

### Electronic supplementary material

Below is the link to the electronic supplementary material.

Supplementary Material 1

Supplementary Material 2

## Data Availability

The datasets used and/or analyzed during the current study are available from the corresponding author on reasonable request.

## Abbreviations

IAV: Influenza A virus
dpi: Days post infection
BALF: Bronchoalveolar lavage fluid
RNA-Seq: RNA Sequencing
CXCR2: C-X-C motif chemokine receptor 2
NLRP3: NLR family pyrin domain containing 3
IL-1β: Interleukin 1 beta
LD50: 50% Lethal dose
PBS: Phosphate buffered saline
p.o.: Oral gavage
i.p.: Intraperitoneally injected
CON: Control
qRT-PCR: Quantitative real-time PCR
H&E: Hematoxylin-eosin
FPKM: Fragments per kilobase millon mapped reads
DEGs: Differentially expressed genes
SPR: Surface plasmon resonance
GAPDH: Glyceraldehyde-3-phosphate dehydrogenase
ASC: Apoptosis-associated speck-like protein containing a CARD
HA: Hemagglutinin
IL-2: Interleukin 2
IL-3: Interleukin 3
IL-5: Interleukin 5
IL-9: Interleukin 9
IL-12: Interleukin 12
IL-13: Interleukin 13
IL-17: Interleukin 17
GM-CSF: Colony stimulating factor 2
G-CSF: Colony Stimulating Factor 3
CCL11: C-C motif chemokine ligand 11
CXCL1: C-X-C motif chemokine ligand 1
CCL3: C-C motif chemokine ligand 3
CCL4: C-C motif chemokine ligand 4
CCL5: C-C motif chemokine ligand 5
CCR3: C-C motif chemokine receptor 3
CCR2: C-C motif chemokine receptor 2
CCR5: C-C motif chemokine receptor 5
KD: Affinity constant
