# Supplementary material for: Phillyrin ameliorates influenza a virus-induced pulmonary inflammation by antagonizing CXCR2 and inhibiting NLRP3 inflammasome activation
Source: Virol J. 2023 Nov 13;20:262. doi: 10.1186/s12985-023-02219-4 (PMC10644626; doi:10.1186/s12985-023-02219-4)
Supplement: Supplementary file 1 — Supplementary Material 1 [file 12985_2023_2219_MOESM1_ESM.docx]

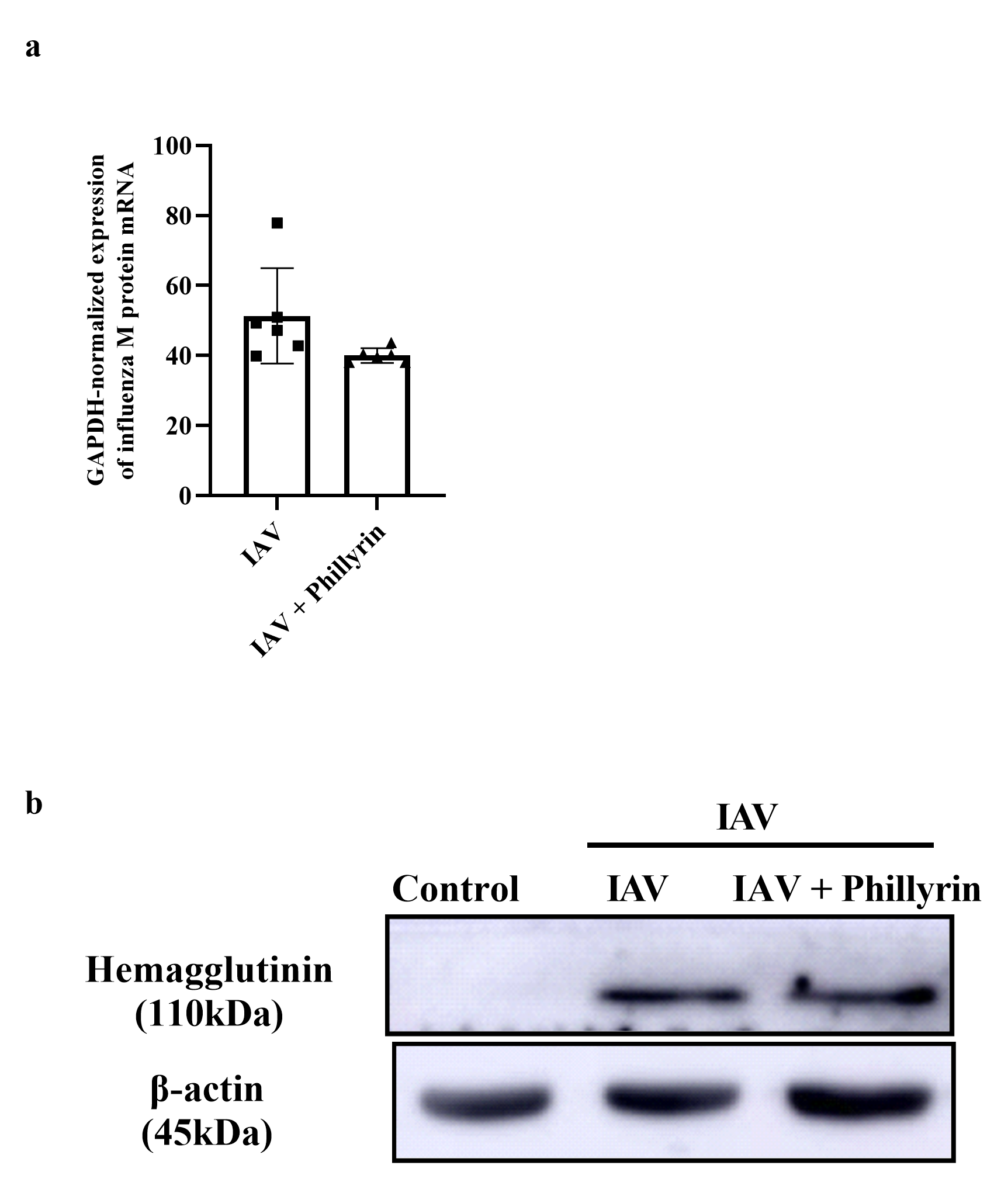


**Fig. S1.** Phillyrin has no obvious inhibition on viral multiplication. (a) On 7 dpi, the mRNA level of influenza virus M protein in the lungs of mice was analyzed by qRT-PCR. Mean±SD (n=6/group). One-way ANOVA, # p<0.05 vs. CON, * p<0.05 vs. IAV. (b) The protein levels of H1N1 hemagglutinin protein in the lungs on 7 dpi were detected by Western blotting.

**
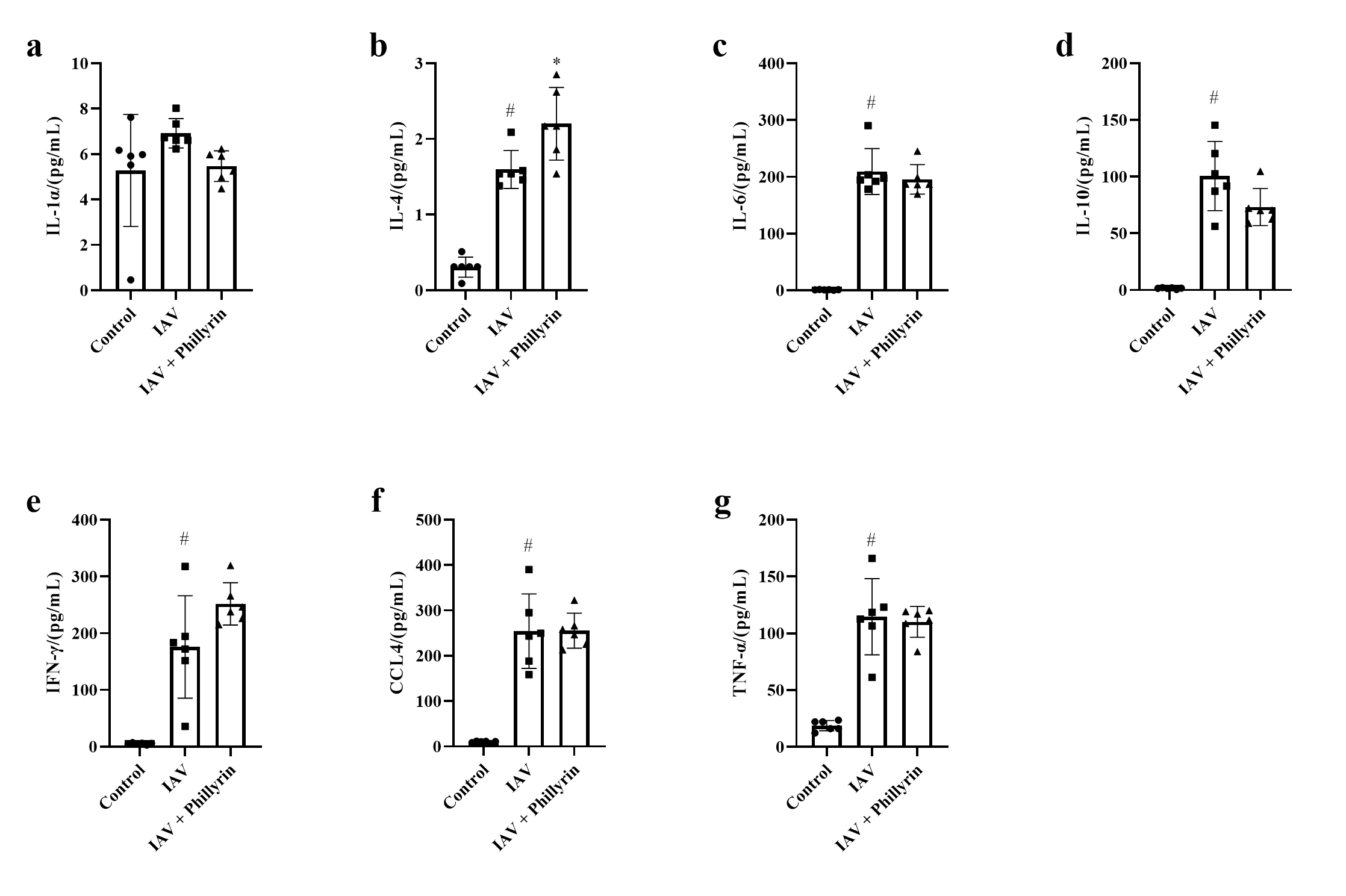
**

**Fig. S2.** The regulation of phillyrin on cytokines in BALF. On 7 dpi, the levels of 23 cytokines in BALF of different treatment groups were detected by Bio-Plex. The figure shows the cytokines with significant changes in secretion level after phillyrin treatment. Mean±SD (n=6/group). One-way ANOVA, # p<0.05 vs. CON, * p<0.05 vs. IAV.

**Table. S1. Primer information**

| Gene Name | Primer | Sequence |
| --- | --- | --- |
| GAPDH (mouse) | GAPDH-F | TGGCCTTCCGTGTTCCTAC |
|  | GAPDH-R | GAGTTGCTGTTGAAGTCGCA |
| CCR1 | CCR1-F | ACTCTGGAAACACAGACTCACT |
|  | CCR1-R | GCCCACCACTCCAATGATGA |
| CCR2 | CCR2-F | AGGAGCCATACCTGTAAATGC |
|  | CCR2-R | GGCAGGATCCAAGCTCCAAT |
| CCR3 | CCR3-F | AAACTTGCAAAACCTGAGAAGC |
|  | CCR3-R | GCCATTCTACTTGTCTCTGGTGA |
| CCR5 | CCR5-F | TAGATTTGTACAGCTCTCCTAGCC |
|  | CCR5-R | CATCCTGCAAGAGCCAGAGTC |
| CXCR2 | CXCR2-F | AAGGGTGGGGAGTTCGTGTA |
|  | CXCR2-R | AGGGAGCAATACTCAGCTTTCA |
| CXCL1 | CXCL1-F | CTGGGATTCACCTCAAGAACATC |
|  | CXCL1-R | CAGGGTCAAGGCAAGCCTC |
| NLRP3 | NLRP3-F | AATGCTGCTTCGACATCTCC |
|  | NLRP3-R | CCAATGCGAGATCCTGACAA |
| ASC | ASC-F | TGAGCAGCTGCAAACGACTA |
|  | ASC-R | CACGAACTGCCTGGTACTGT |
| Caspase 1 | Caspase1-F | GTGGAGAGAAACAAGGAGTGG |
|  | Caspase1-R | AATGAAAAGTGAGCCCCTGAC |
| IL-1β | IL-1β-F | GGTGTGTGACGTTCCCATTA |
|  | IL-1β-R | GGCCACAGGTATTTTGTCGT |
| Influenza M-protein | Influenza M-protein-F | GGACTGCAGCGTTAGACGCTT |
|  | Influenza M-protein-R | CATCCTGTTGTATATGAGGCCCAT |
| GAPDH (dog) | GAPDH-F | AGTCGGAGTGAACGGATTTGG |
|  | GAPDH-R | AGTTGTCATGGATGACCTTGG |
| Influenza nucleo protein | Influenza nucleo protein-F | TCAAACGTGGGATCAATG |
|  | Influenza nucleo protein-R | GTGCAGACCGTGCTAAAA |

**
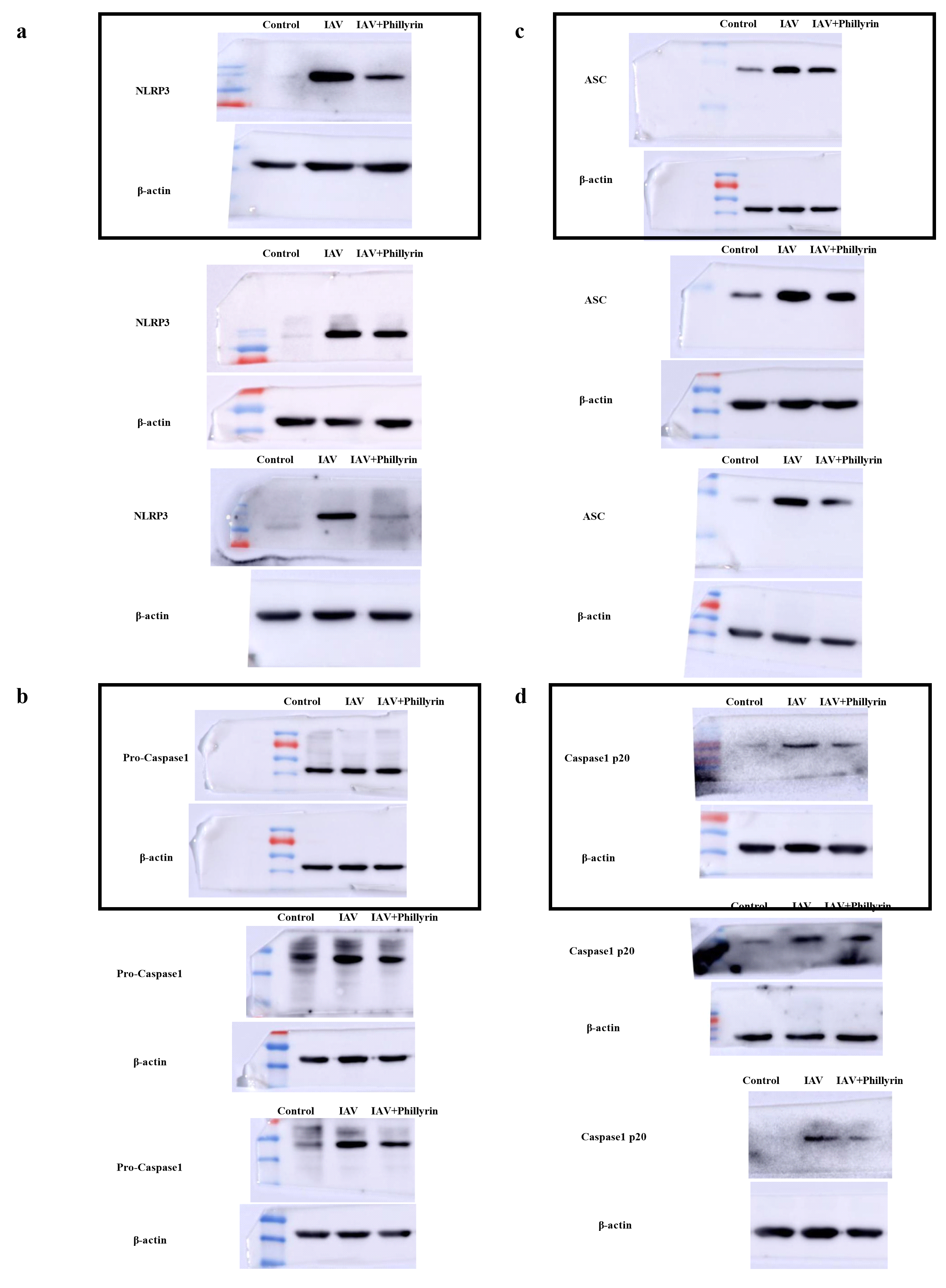
**

**Fig. S3.** The uncropped blot images of NLRP3（a）, Pro-Caspase 1（b）, ASC（c）Caspase 1 P20（d）and corresponding reference proteinβ-actin, respectively. The Western blotting assays of each protein were repeated independently for 3 times.The images marked in the black box are the original image of the cropped blot images shown in Fig.7.β-actin was employed as the reference protein and the relative values of the target proteins of all images were calculated with ImageJ software.

**
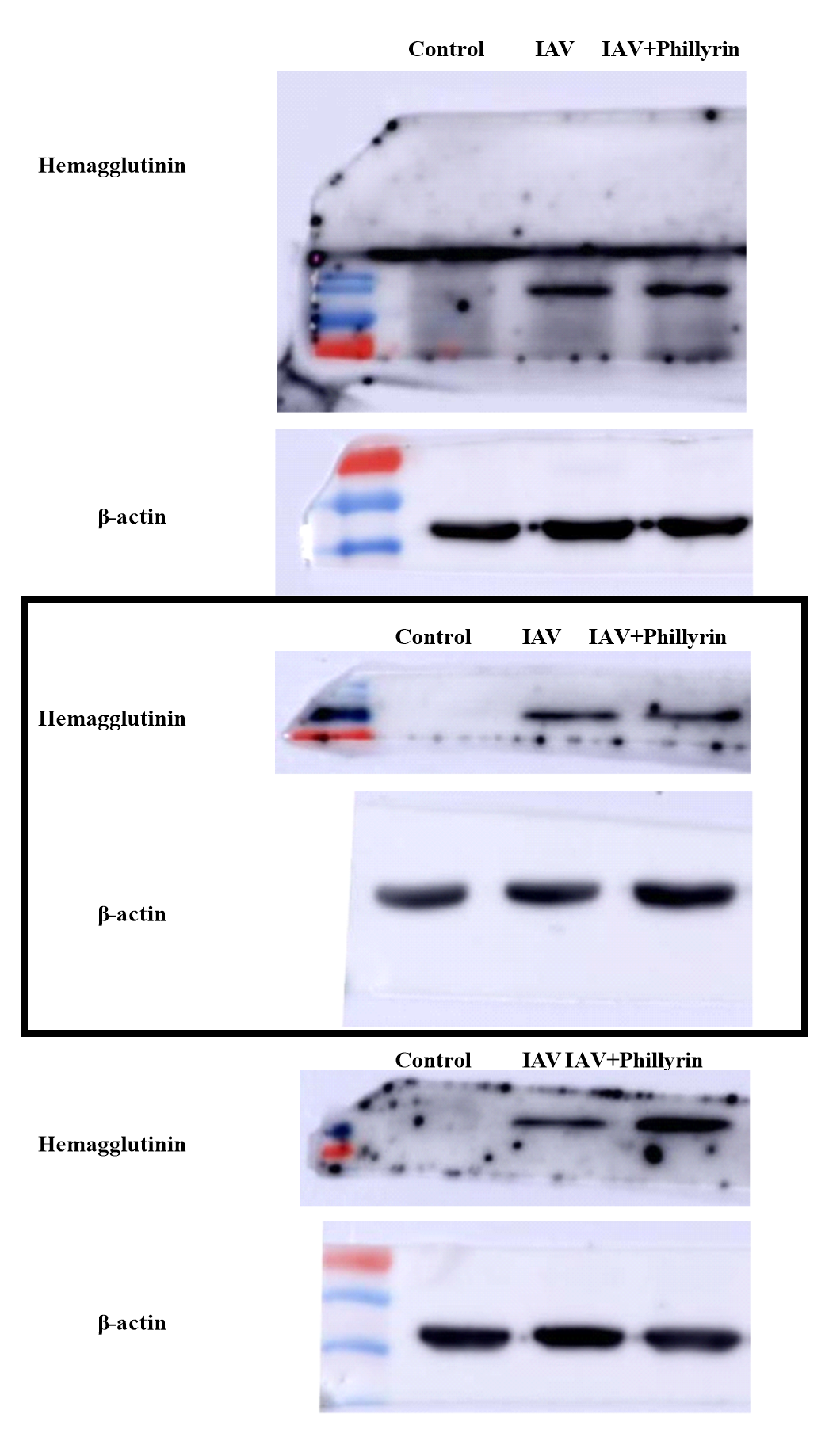
**

**Fig. S4.** The uncropped blot images of HA protein and corresponding reference proteinβ-actin . The Western blotting assays of each protein were repeated independently for 3 times.The images marked in the black box are the original image of the cropped blot images shown in Fig.S1.
